# Gambling Disorder and suicide risk – a clinical case

**DOI:** 10.1192/j.eurpsy.2023.1191

**Published:** 2023-07-19

**Authors:** P. Tirlea, R. Tipa, I. Luciana

**Affiliations:** 1 Alexandru Obregia clinical Psychiatry Hospital; 2 Carol Davila University of Medicine and Pharmacy, Bucharest, Romania

## Abstract

**Introduction:**

Pathological gambling is an addictive disorder and a current important issue with substantial social and personal costs. It is associated with impaired functioning, criminal record, bankruptcy and mental health problems. There is a significant comorbidity between gambling disorder, mood disorders and other addictive behaviors like alcohol use. Suicidality is common, impulsivity being a major risk factor for suicidal acts.

**Objectives:**

Case presentation of gambling disorder associated with a suicide attempt

**Methods:**

Review of the clinical file of a patient diagnosed with gambling disorder and non-systematic review on the topic on PubMed

**Results:**

A 35 old male patient is brought to our psychiatrical emergency unit by means of ambulance as he attempted to commit suicide by inflicting multiple deep cuts on his forearms. He has a positive history of gambling disorder, no prior suicide attempt, or criminal record. He has a precarious economic status, the trigger for his acts being the loss of a substantial financial amount. The risk factors in his case were a positive familial history of addictive disorders (his father was diagnosed with alcohol use disorder), aversive childhood events, comorbid depression, alcohol misuse and low income. The patient resumed his gambling behavior 7 months prior to admission, after a 5 year abstinence, motivated by the desire to rapidly pay a loan he recently took. The addictive behavior worsened after his wife experienced a miscarriage. He started borrowing money, engaging in antisocial acts like stealing money from his wife’s bank account, neglecting his job and ending up in financial debt. He experienced feelings of alienation and isolation from his social network and family, unable to verbalize his burden. He also feared a divorce. Psychological coping strategies such as thought and emotional suppression were present and also an important tendency to minimize the severity of the events. Cluster B traits were present but not clinically significant. The suicide attempt is described by the pacient as being impulsive, with no prior planning, as a mean of problem solving for his desperate situation of high financial and social burden.

In the hospital setting, pharmacological treatment with SSRI Escitalopram and opiate antagonist Naltrexone was initiated. The patient was referred to psychological counseling during hospitalisation and to CBT after he left the hospital. He had excellent social support.

**Conclusions:**

Although suicide is initially seen as an impulsive act, in fact it includes a constellation of thoughts, emotions and behaviors which lead to the hopelessness and desperation preceding the suicidal attempt. Gambling disorder tends to have a chronic evolution, impacting many important life domains, complex management such as pharmacotherapy, psychological interventions and social support being necessary for a favorable outcome.

**Disclosure of Interest:**

None Declared

